# Hardware‐Native Gaussian Sampling With Probabilistic Ising Machines for Modern Communication Paradigms

**DOI:** 10.1002/advs.78214

**Published:** 2026-10-11

**Authors:** Davi Rodrigues, Andrea Grimaldi, Deborah Volpe, Youwei Bao, Shuhan Yang, Eleonora Grassucci, Giulio Siracusano, Massimo Chiappini, Mario Carpentieri, Hyunsoo Yang, Giovanni Finocchio

**Affiliations:** ^1^ Department of Electrical and Information Engineering Politecnico di Bari Bari Italy; ^2^ Istituto Nazionale di Geofisica e Vulcanologia Rome Italy; ^3^ Department of Electrical and Computer Engineering National University of Singapore Singapore Singapore; ^4^ Department of Information Engineering Electronics and Telecommunications Sapienza University of Rome Rome Italy; ^5^ Department of Mathematical and Computer Sciences Physical Sciences and Earth Sciences University of Messina Messina Italy

**Keywords:** edge generative AI, gaussian sampling, post‐quantum cryptography, probabilistic ising machines, semantic communication, spintronic computing

## Abstract

Modern communication systems increasingly shift computation to edge devices, where post quantum signing, generative AI and semantic communication must operate under strict energy, latency and memory constraints. A central bottleneck in these workloads is Gaussian sampling. In Falcon class signatures, sampling accounts for about 25% of operations and about 65% of signing time. In diffusion inference, repeated Gaussian injection across denoising steps makes edge deployment costly. Here we show that probabilistic Ising machines provide a hardware native route to random Gaussian distributions with both tunable variance and sampling density with two independent parameters. We compare fully digital field‐programmable gate array (FPGA) and FPGA‐spintronic implementations with software implementation. At matched sampling precision, the proposed FPGA sampler reduces the dynamic power and logic utilization of the Gaussian‐sampling core by approximately 4.0‐fold and 4.4‐fold, respectively, relative to the isolated Falcon core. A projected ASIC implementation in 28 nm CMOS is estimated to consume approximately 8 mW. Image generation quality quantified with well‐established metrics is preserved in an open‐source diffusion pipeline. These results establish probabilistic Ising machines as an efficient and scalable platform for Gaussian sampling in post quantum cryptography, edge generative inference and semantic communication.

## Graphical abstract

Probabilistic Ising machines are introduced as hardware‐native Gaussian samplers for edge communication workloads. Tunable variance and sampling density enable accurate distributions for Falcon‐class post‐quantum signatures and diffusion‐based semantic communication. FPGA and spintronic implementations reduce energy, logic cost, and latency while preserving image‐generation quality, establishing probabilistic hardware as a scalable sampling primitive.

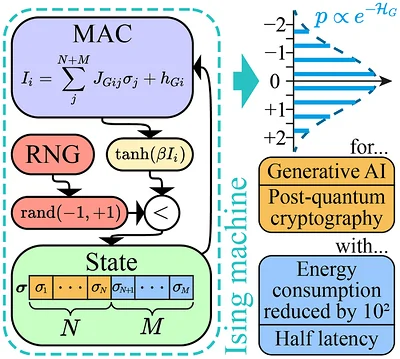

## 1 Introduction

Modern communication paradigms demand new methods to scale the data volumes, reliability targets, security, privacy requirements, and latency constraints anticipated for 6G services such as immersive telepresence, digital twins, smart healthcare, industrial automation, and autonomous platforms [1, 2, 3, 4]. This is driving the joint adoption of semantic communication and quantum‐resistant cryptography. Semantic communication reduces communication load by transmitting task relevant representations instead of raw data. Post quantum cryptography (PQC) preserves security against quantum capable adversaries. In both cases, a major shift of the performance burden moves toward edge and Internet of Things (IoT) devices, where on‐device processing is preferred for privacy, resilience, and latency, but where energy, memory bandwidth, and silicon area remain severely constrained [1, 2, 3, 4, 5, 6].

One of the central building blocks across these workloads is the Gaussian sampling [7, 8, 9, 10]. In diffusion models, generation begins from a standard Gaussian distribution and proceeds through iterative denoising with Gaussian transitions at every step [11, 12]. In semantic communication, the receiver‐side decoder must therefore sustain repeated noise driven updates while preserving semantic structure under channel degradation [6, 13]. This makes sampling fidelity and sampling throughput direct determinants of latency, energy per output and reconstruction quality and remain central obstacles for deployment at the edge [6, 7, 11, 12, 14, 15, 16, 17].

The same primitive is fundamental in lattice‐based post‐quantum signatures. For example, in Falcon [9, 10, 18, 19] optimized implementations, Gaussian sampling dominates signing cost and can account for about 65% of total computational costs [9, 10, 19]. Across both generative AI and post‐quantum cryptography, Gaussian generation therefore sets a key trade‐off among statistical correctness, robustness, latency and energy. Hardware‐native randomness generation is thus a system‐level bottleneck for the quality of edge diffusion inference and for the energy consumption of Falcon‐class signature engines, directly constraining energy per sample, end‐to‐end latency and, ultimately, the model or security budget that can be sustained on device.

Probabilistic Ising machines (pIMs) are emerging as an efficient hardware paradigm for computation, with demonstrated use in Boltzmann‐machine emulation and combinatorial optimization [20, 21, 22]. They are especially well suited to sampling intensive workloads. Sparse pIMs support massively parallel stochastic updates, with reported throughput gains of up to six orders of magnitude relative to Central Processing Unit (CPU) Gibbs sampling and about 5‐fold to 18‐fold relative to Graphics Processing Unit (GPU) and Tensor Processing Unit (TPU) implementations [21]. They can also accelerate mixing and decorrelation, with projected end‐to‐end speedups of 10^2^‐fold to 10^4^‐fold in sampling‐dominated workloads [8]. At the circuit level, compact probabilistic architectures have achieved random‐bit generation near 10 MHz with energy near 1 fJ per bit and system‐level energy reductions of about four orders of magnitude per generated sample relative to GPU baselines [8, 23, 24, 25]. They are also compatible with spintronic devices such as magnetic tunnel junctions (MTJ), which offer compact, energy‐efficient and high‐quality sources of stochasticity [20, 26, 27, 28, 29]. These attributes make pIMs a strong hardware candidate for edge post‐quantum cryptography and generative inference, where sampling cost strongly constrains energy and memory bandwidth.

In this work, we introduce pIM‐based hardware Gaussian samplers for post‐quantum cryptography and edge diffusion inference, and experimentally validate their use in diffusion‐based semantic communication workflows. We consider a full digital implementation on field‐programmable gate array (FPGA) and a digital/analog implementation combining FPGA with the switching characteristics of thermally stable spin transfer torque MTJ (STT‐MTJ). By programming local biases and couplings, the sampler provides tunable variance, controllable sampling density and accurate tail generation, reaching tail accuracy up to 7.5σ with only four Ising nodes while we can set the resolution in this range as large as we want by adding additional Ising spins. The underlying algorithm relies on simple hardware primitives, which enables efficient implementation and straightforward scaling across platforms. Because pIMs generate samples with probabilities already shaped by the target distribution, they can also reduce latency by suppressing much of the rejection overhead that limits conventional Falcon Gaussian samplers, while further acceleration becomes possible through massively parallel stochastic updates. At the device level, the MTJ sampling core operates at about 0.02 mW, around two orders of magnitude below the corresponding fully digital random‐number‐generation block, although the system‐level energy is still dominated by peripheral circuitry and external Multiply‐and‐Accumulate (MAC) operations. In Falcon‐class PQC hardware, the proposed sampler reduces projected application‐specific integrated circuit (ASIC) power to about 8 mW, compared with 62 mW for state‐of‐the‐art implementation. It also improves hardware efficiency, using about 4.4times fewer logic slices than the reference Gaussian core and avoiding Digital Signal Processing (DSP) blocks, which are among the most valuable and constrained computational resources in FPGA fabrics. Across a systematic multi‐metric evaluation, the proposed samplers achieve statistical fidelity comparable to established digital baselines while substantially reducing power and hardware cost. These results establish pIM‐based Gaussian sampling as a practical and scalable hardware primitive for secure and efficient edge intelligence.

## 2 Gaussian Distribution Sampling Based on Ising Machines

pIMs provide a hardware native route to Gaussian sampling with favourable energy and area scaling, because they exploit intrinsic probabilistic dynamics rather than arithmetic intensive transformations [8, 20, 21, 30, 31, 32]. The Ising energy landscape is defined by the quadratic Hamiltonian $H\left(\sigma \right)=-\frac{1}{2}\sum_{i,j}J_{\mathrm{ij}}\sigma_{i}\sigma_{j}-\sum_{i}h_{i}\sigma_{i}$, where $\sigma_{i}\in \left\{-1,+1\right\}$ are stochastic spins, ***J***_***ij***_ are coupling coefficients, and ***h***_***i***_ are local biases. Under probabilistic operation, the machine evolves toward a stationary distribution proportional to **exp**(−***H***), enabling direct sampling from a target Boltzmann distribution. Prior approaches have leveraged this property to approximate Gaussian statistics, but they often require additional continuous parameters, nonuniform discretization, or complex calibration to control variance and tail behaviour [25, 32, 33, 34]. Here we implement directly in hardware a compact and analytically tractable mapping that provides direct control of the generated distribution, including variance, tail accuracy, and sample density.

We encode a real valued sample ***x*** using a set $\sigma =\{\sigma_{1},\text{…},\sigma_{N+M}\}$ of *N + M* stochastic spins, where the first *N* spins set the dynamic range and the remaining *M* spins refine the sampling density. Specifically, we define

(1) $$x=\sum_{i=1}^{N}2^{\;i-2}\;\sigma_{i}+\sum_{i=1}^{M}2^{-1}\left(12^{-i}\right)\sigma_{i+N}.$$

By choosing an Ising Hamiltonian ***H***_***G***_(***x***) = ***x*** ^2^/2, the resulting stationary distribution over ***x*** approaches a standard normal distribution with zero mean and unit variance, with deviations governed by the finite discretization induced by *N* and *M*. The construction generalizes to arbitrary mean µ and standard deviation ***s*** by shifting and scaling the energy as ***H***_***G***_(***x***; µ, ***s***) = (***xμ***)^2^/(2 ***s*** ^2^), which preserves the Boltzmann form while directly setting the target moments. Figure 1a illustrates the encoding from spin configurations to real valued samples for *N* = 2 and *M* = 1, while Figure 1b,c show how *N* determines the attainable range and therefore tail accuracy, and how *M* controls the sampling density within that range. The parameter *N* sets the range by uniformly expanding the set of representable values with a fixed spacing of 1, yielding a support of [−(2^***N***^ − 1)/2, (2^***N***^ − 1)/2], while retaining the target standard deviation. We notice that for *N* = 4, we theoretically obtain a tail accuracy of 7.5σ. The parameter *M* increases sampling density by inserting additional representable values between these integer points, reducing the spacing by a factor of 2 *^M^*.

**FIGURE 1:**
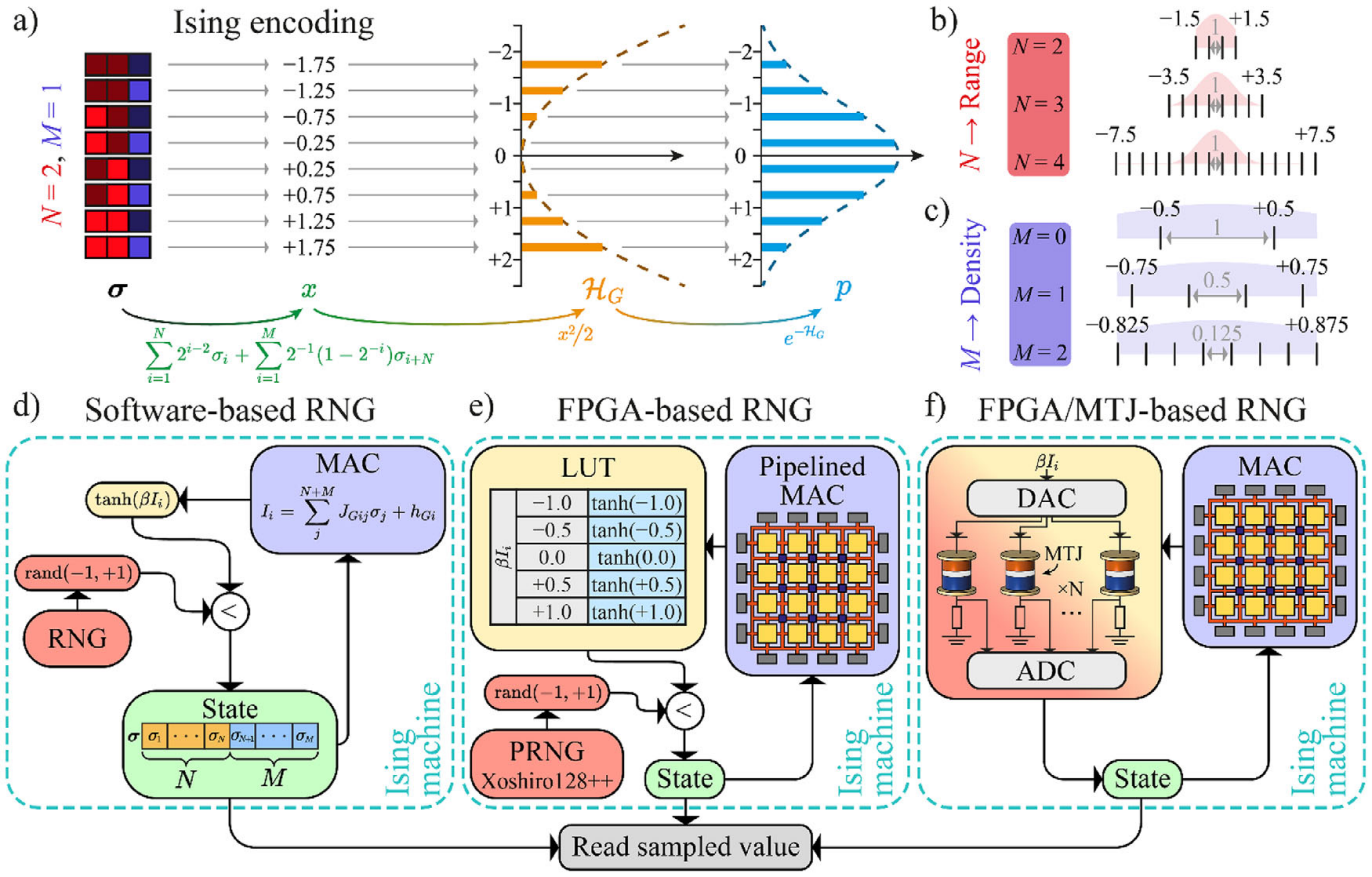
Tunable Ising Mapping for Gaussian distribution generation: (a) Mapping of a target Gaussian distribution onto an Ising Hamiltonian. The distribution parameters are encoded into coupling weights and local biases, resulting in a Boltzmann distribution over spin configurations. Each configuration of the *N+M* spins corresponds to a discrete value of the sampled distribution, with occurrence frequency governed by the target Gaussian statistics. (b,c) Schematic illustration of the tunability of the generated Gaussian distribution as a function of parameters *N* and *M*, respectively. These parameters independently control the tail accuracy and the sampling density of the distribution. (d–f) Comparison of Ising machine realizations based on software, FPGA, and FPGA/MTJ hardware. All implementations share a common architecture comprising random number generation, state update via Multiply‐and‐ACcumulate (MAC) operations, and stochastic spin dynamics, while differing in how nonlinearity and randomness are realized. DAC and ADC represent the Digital‐to‐Analog and Analog‐to‐Digital converters.

PIM algorithm reduces to three primitives, (i) linear MAC operations, (ii) evaluation of a hyperbolic tangent (*tanh*) nonlinearity, and (iii) random number generation (RNG). These primitives can be mapped to specialized hardware blocks with different efficient trade‐off. Figure 1d–f compares three realizations of Gaussian random generation based on pIM deployed as (d) full software baseline, (e) a fully digital FPGA implementation, and (f) a digital‐analog FPGA/MTJ implementation. In the hardware realizations, the MAC is executed on the FPGA to exploit parallel arithmetic and high throughput, operating at 100–200 MHz. In a full digital implementation, the nonlinearity and sampling are typically realized with a LookUp Table (LUT) for the *tanh* calculation and a pseudorandom number generator (PRNG), which incur substantial memory traffic and logic overhead. The digital‐analog architecture replaces these blocks with the switching probability of MTJs that provide an effective *tanh*‐like response and intrinsic randomness at the device level [35, 36], thereby reducing digital complexity for both operations. Because the accumulation remains digital, interfacing the MTJ array with the FPGA requires digital‐to‐analog (DAC) and analog‐to‐digital (ADC) stages. Implementation details are reported in the Methods sections “FPGA implementation” and “FPGA/MTJ implementation”.

The key metrics for assessing the advantages of pIMs are (i) energy consumption, (ii) area footprint and (iii) Gaussian distribution quality. First, we estimated the energy cost of Gaussian generation for both FPGA and FPGA/MTJ realizations. Table 1(a) reports the estimated power consumption the FPGA based and MTJ based implementations employing *N* = 4 and *M* = 0, estimated following the methodology described in the Methods section “FPGA resource utilization and power estimation”. In both designs, the MAC block is implemented on the FPGA and consumes approximately 1 mW, consistent with the efficiency of FPGA fabrics for parallel MAC operations. In the fully digital realization, the *tanh* is computed using an optimized approach combining linearization, a LUT and saturation, where the LUT is synthetized as read‐only memory with an associated power cost of about 1 mW. The dominant power contribution is the Xoshiro128++ pseudorandom number generator at 7 mW, reflecting the cost of producing high quality randomness in deterministic digital circuitry. In the digital‐analog realization, the MTJs provide both an effective *tanh* response and intrinsic randomness directly, with a measured consumption of 0.02 mW, roughly two orders of magnitude lower than the digital PRNG block. The system level budget, however, is set by the interface, since the DAC and ADC stages required to couple the MTJ array to digital logic consume approximately 10 mW per MTJ. Consequently, while the MTJ core offers an intrinsic two order of magnitude reduction for the nonlinearity and sampling primitives, the end‐to‐end advantage depends on reducing converter overhead through tighter mixed signal integration and converter co‐design.

**TABLE 1:** Energy and area efficiency: (a) Estimated power consumption in milliwatts for FPGA‐based and MTJ‐based implementations, decomposed into MAC, hyperbolic tangent, random number generation, and converter overhead. (b) Resource usage summary for representative Gaussian sampling implementations, including the FPGA based and FPGA/MTJ based realizations reported in this work. ^1^Synthesis targeting a ZCU106 device. Most of the memory cost is due to the storage of hyperbolic tangent values (45 kb). The LookUp Table (LUT)‐based implementation can be replaced with alternative hardware solutions to achieve area reduction at the cost of increased latency. ^2^Synthesis targeting a VCU118 device. Only the computational area is reported to ensure a fair comparison with other implementation.

a) Estimated power consumption (mW)			
FPGA implementation	FPGA		
	MAC	tanh	RNG
	1	1	7
FPGA/MTJ implementation	FPGA	MTJ board	
	MAC	tanh + RNG	
		DAC + ADC	MTJ
	2	10	0.02

b)	Logic slices/CLB	BlockRAM (kb)	DSP	Bit width
This work FPGA (N = 4)^1^	450	72	0	32I
This work FPGA/MTJ (N = 4)^2^	323	18	0	27I

Second, we evaluated the area footprint and FPGA resource utilization. Table 1(b)) summarizes the FPGA resource utilization and highlights the complementary trade‐offs between the two hardware implementations [35, 36, 37, 38, 39, 40, 41, 42]. “Logic slices/Logic CLB” indicates the use of Configurable Logic Blocks (CLBs) in the architectures, which implement the combinational and sequential logic required for data processing and control “BlockRAM” reports the on chip embedded memory allocated, in kilobits (kb), typically used for LUTs, buffering, and interface storage. “DSP” denotes the number of dedicated Digital Signal Processing (DSP) blocks used to implement hardware multiplication and other complex arithmetic operations. “Bit width” specifies the fixed‐point precision used for representing the generated Gaussian random numbers and directly affects both resource usage and numerical fidelity.

Our FPGA‐based pIM implementation was evaluated at 32‐bit integer precision, requiring 450 logic slices, respectively, together with only 72 kb of Block RAM. These resource requirements indicate a competitive embedded‐memory footprint. The FPGA/MTJ‐based implementation, operating at 27‐bit integer precision and presently limited by peripheral circuitry, requires 323 logic slices and 18 kb of BlockRAM. Notably, neither implementation uses DSP slices, preserving dedicated complex operation resources for other tasks and facilitating co‐integration on shared FPGA fabrics. Overall, both realizations combine state‐of‐the‐art memory footprint with enhanced precision, providing a practical hardware profile for PQC dedicated hardware.

Third, we evaluated the Gaussian distribution quality. Figure 2 shows the sampled probability mass functions obtained with (a,b) a software based generator, (c,d) an FPGA based Ising machine, and (e,f) a FPGA/MTJ based Ising machine. The logarithmic plots in Figure 2a,c,e show how the increasing of *N* extends the representable range improving the tail resolution. In contrast, Figure 2b,d,f show how *M* refines the distribution by increasing sampling density, without changing the tail resolution. The software and FPGA based implementations are quantitatively consistent in both the central region and the tails, consistent with their shared use of deterministic arithmetic driven by pseudorandom inputs. The MTJ based implementation closely matches the target distribution in the high probability region but exhibits a small deviation in the extreme tails, which we attribute to device‐to‐device differences. This deviation is not a fundamental limit and can be reduced through calibration and tighter control of operating conditions. For further details on the quality of the Gaussian sampling, see Note S1.

**FIGURE 2:**
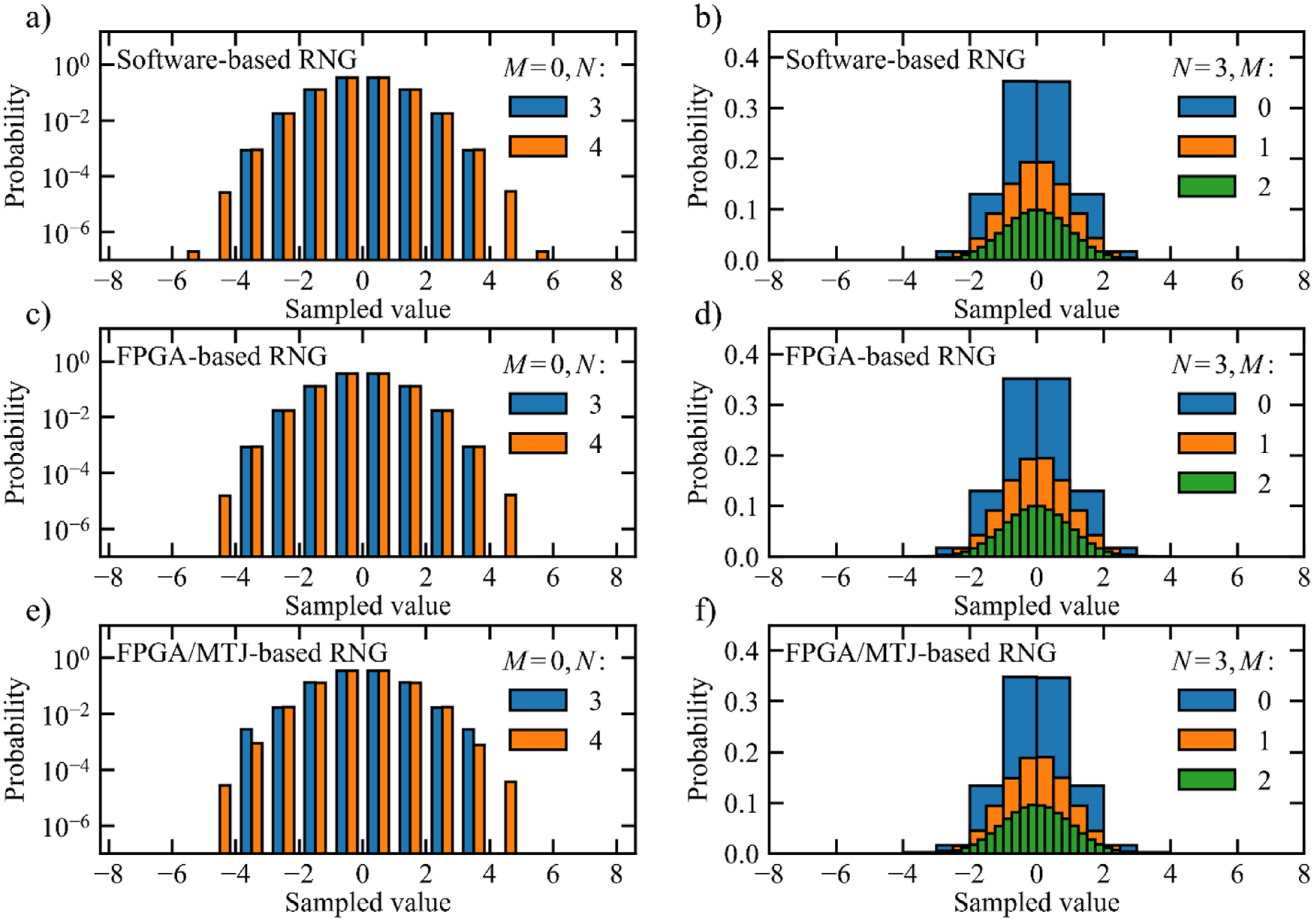
Gaussian distribution generation: Comparison of Gaussian distributions generated using software based sampling (a,b), FPGA based Ising machine sampling (c,d), and FPGA/MTJ based Ising machine sampling (e,f). Parameter *N* controls the dynamic range and tail coverage (a,c,e), and parameter *M* controls the sampling density (b,d,f). The vertical axis in the left column is logarithmic to emphasize the tails. The MTJ based case shows a small tail deviation that is consistent with sensitivity to experimental calibration and device nonidealities.

In the following sections, we evaluate the relevant key metrics against the implementation constraints imposed by PQC and diffusion models.

## 3 Energy Efficiency for Post‐Quantum Cryptography

Falcon is a lattice‐based post quantum signature scheme built on N^th^ degree Truncated Polynomial Ring Units (NTRU) lattices [9, 10, 19, 37]. Security is defined over structured polynomial rings, which contributes to compact public keys and signatures and supports efficient verification. These properties are attractive for systems with tight bandwidth, storage and authentication latency constraints. Signature generation is substantially more demanding. It combines fast Fourier transforms, split and merge operations, recursive fast Fourier sampling and double precision floating point arithmetic within a single execution flow.

Figure 3a summarizes the Falcon signature‐generation workflow. Falcon signing first hashes the message with the public key to define a target point in the NTRU lattice, and then uses the private trapdoor to sample a short lattice vector from a discrete Gaussian distribution centred on that target. The sampled vector is recovered, checked against the norm bound, and, if valid, encoded as the output signature. Sampling energy is a key metrics in Falcon hardware. Each signature requires 1024 Gaussian samples in Falcon‐512 and 2048 in Falcon‐1024 [9, 19, 37]. In optimized hardware software co‐design, sampling accounts for about one quarter of total execution time [9, 10]. In full hardware accelerators, their contribution is markedly larger and has been reported at about 65% of total signing time [9]. The same class of implementations reports average power near 65 mW and energy per signature in the range of about 20 to 30 µJ [9]. Therefore, Gaussian sampling is the main target for reducing both area and energy in Falcon class accelerators.

**FIGURE 3:**
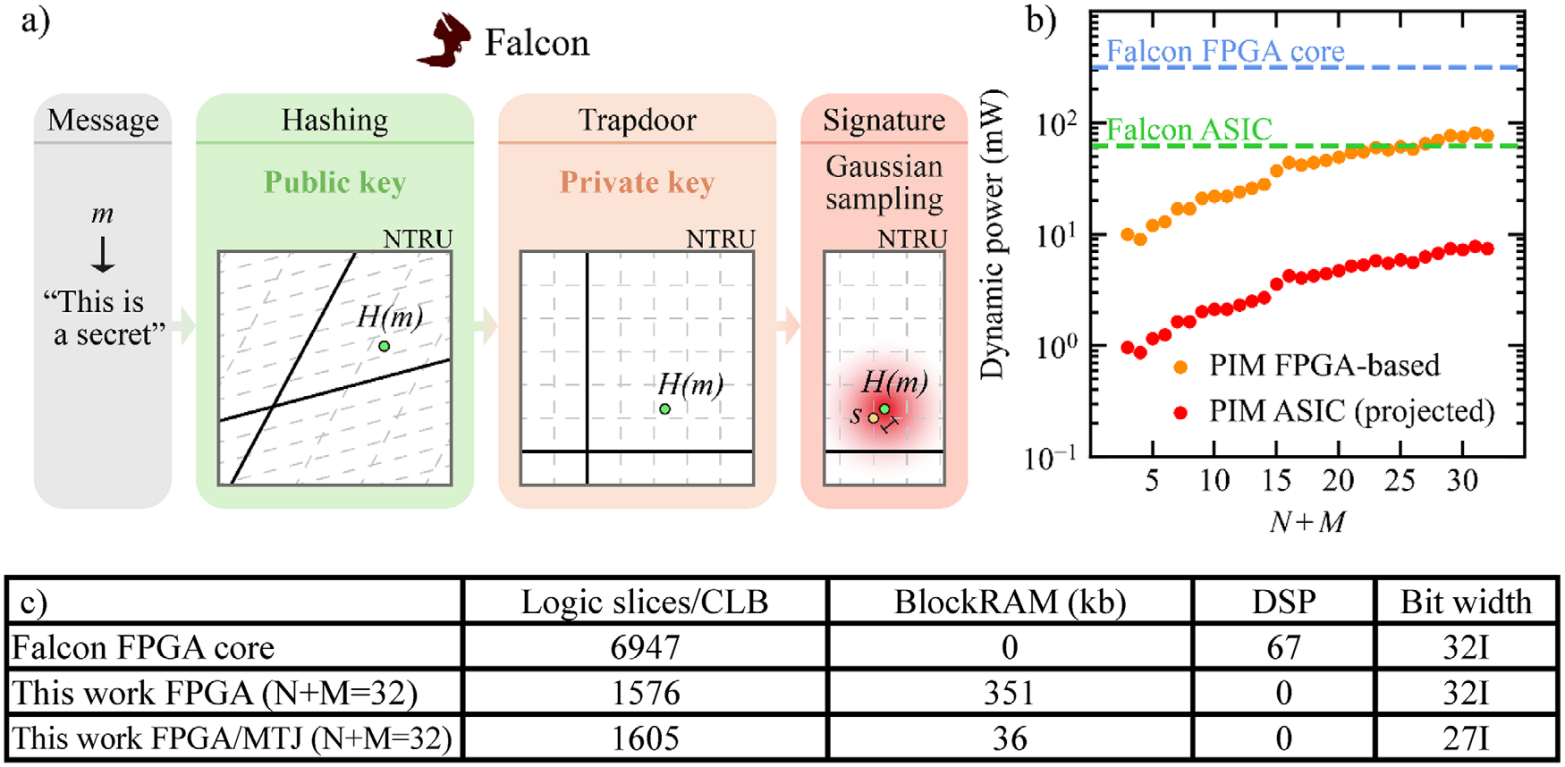
Falcon signing and hardware impact of the proposed sampler. (a) Overview of the Falcon signing flow. The message ***m*** is first hashed and mapped, through the public‐key‐defined NTRU lattice structure, to a target point ***H*** (***m***). Using the private trapdoor, Falcon then performs discrete Gaussian sampling to generate a short lattice vector centered on that target. The sampled vector is recovered and checked against the norm bound to produce the output signature. (b) Dynamic power comparison between a state‐of‐the‐art hardware‐optimized Falcon sampler [9] and the proposed pIM‐based realization as a function of the total number of Ising nodes, *N* + *M*. The plotted Falcon reference corresponds to the isolated Gaussian‐sampling core of [9] (sampling arithmetic only), while the complete Z‐sampler, which additionally includes the ChaCha20 cryptographic PRNG and the floating‐point conversion units, is reported in the text as a secondary, system‐level reference. The reported results include both the dynamic power estimates obtained on the FPGA implementation (ZCU106 board) and a comparison with the 28 nm ASIC values reported in [9]. Additionally, a projected power consumption of the proposed architecture at 28, nm is obtained by scaling the FPGA measurements using the FPGA‐to‐ASIC power ratio reported for the Falcon sampler. (c) Resource utilization comparison between the proposed FPGA pIM implementation and the isolated Falcon Gaussian core of ref. [9] (sampling arithmetic only, excluding the ChaCha20 PRNG and the floating‐point conversion units), while the corresponding values for the complete Z‐sampler are reported in the text. The proposed design substantially reduces logic and DSP usage while maintaining a modest BlockRAM footprint, highlighting the efficiency of its simple hardware‐native primitives. Synthesis performed targeting a ZCU106 board.

Figure 3b compares the dynamic power of the two designs of the pIM‐based Gaussian sampler with Falcon hardware sampler implementation described in ref. [9]. We report the power consumption of the Gaussian‐sampling core alone, excluding the pseudorandom‐number generator, whose power requirements depend strongly on the selected entropy source and its security specifications. The comparison is plotted as a function of the total number of Ising nodes, *N* + *M*, which sets the sampling density and tail fidelity of the generated distribution. At *N* + *M* = 32, matching the bit accuracy of the reference Falcon implementation, the pIM FPGA sampler consumes approximately 80 mW. The isolated Falcon Gaussian‐sampling core consumes about 316 mW, whereas the complete Z‐sampler consumes about 641 mW, including the ChaCha20 cryptographic pseudorandom number generator and floating‐point conversion units. Comparison with the functionally matched Gaussian core, after excluding these two blocks from both implementations, shows an approximately fourfold reduction in power consumption. Comparison with the complete Z‐sampler yields an eightfold reduction, which also reflects the power costs of cryptographic random‐number generation and data conversion. The pIM FPGA result is also comparable to the reported Falcon ASIC value of about 62 mW. Although FPGA and ASIC power values are not directly comparable, this comparison remains informative because the proposed FPGA sampler already reaches the power range of a dedicated ASIC realization of the conventional design. The projected pIM ASIC implementation further reduces the power to about 8 mW, preserving an approximately one‐order‐of‐magnitude advantage when equivalent implementation technologies are compared. This projection is obtained by scaling the FPGA‐based measurements using the FPGA‐to‐ASIC power ratio reported for the Falcon sampler [9].

Figure 3c shows that the power advantage is accompanied by a marked change in hardware cost. The reference Falcon implementation [9] requires 6947 logic slices for the Gaussian core and 13 676 slices for the full sampler design, together with 67 and 74 DSP blocks, respectively. By comparison, the FPGA pIM implementation at *N+M =* 32 uses 1576 logic slices and no DSP blocks, with 351 kb of BlockRAM. The FPGA/MTJ implementation uses 1605 logic slices, no DSP blocks and only 36 kb of BlockRAM. Relative to the full Falcon sampler, this corresponds to an 8.7‐fold reduction in logic slices. Relative to the isolated Gaussian core, the reduction remains 4.4‐fold. The elimination of DSP usage is important because DSP blocks are limited resources in FPGA fabrics and are often reserved for arithmetic intensive tasks elsewhere in the system. The gap between the Falcon Gaussian core and the full sampler also quantifies the hardware cost of conventional random number delivery, since the full design includes the ChaCha pseudorandom generator and interface logic. The proposed sampler retains its power and area advantage in both deployment modes, whether it is coupled to an external cryptographically secure uniform source on FPGA or to an intrinsic physical entropy source in the spintronic implementation.

This point is especially relevant for PQC, where the entropy source is a first‐class hardware concern. Secure samplers are driven by validated uniform random‐number generators and therefore inherit the overhead of entropy generation, conditioning and health testing. Conventional FPGA and CMOS TRNGs usually extract entropy from jitter or metastability in timing‐sensitive circuits [38, 39, 40], with representative CMOS realizations reporting 23 pJ bit^−1^ at 23 Mb s^−1^ in 375 µm^2^, or 3 pJ bit^−1^ in 4,000 µm^2^ [38, 39, 40, 41, 42]. MTJ devices offer a more compact physical source of stochasticity. FPGA‐actuated MTJ systems have generated more than 10^12^ true random bits at rates above 10 Mb s^−1^, with XOR‐conditioned output passing the full NIST statistical test suite [43]. VCMA‐based MTJs further enable on‐demand random‐bit generation in less than 10 ns per bit, support projected single‐device rates close to 100 Mb s^−1^ with fast readout, and have been projected to operate at 10 Mb s^−1^ with an energy cost of 0.43 pJ per bit in integrated probabilistic hardware [44]. More broadly, MTJs provide a highly efficient entropy source for secure hardware, with the dominant residual energy and area costs shifting from entropy generation itself to the surrounding analogue readout and conversion circuitry. In the proposed framework, the sampler can therefore interface with standard cryptographic entropy sources or exploit intrinsic device‐level stochasticity in MTJ hardware while preserving the gains in power and hardware efficiency.

pIMs also provide a latency advantage because Falcon uses rejection‐based discrete Gaussian sampling. In the conventional Falcon sampler, integer candidates are drawn from an auxiliary distribution and accepted only after a correction step that recovers the exact target law. For Falcon parameters, the acceptance probability is close to one half, so each valid sample requires about two candidate draws on average. This rejection overhead increases both latency and random‐number demand across the full signing flow. A pIM‐based sampler reduces this inefficiency by generating values with probabilities already shaped by the target distribution, which can reduce sampling latency by about 2‐fold at the algorithmic level. Further gains arise from the hardware architecture. Sparse pIMs support massively parallel stochastic updates, with flip rates that scale approximately linearly with the number of probabilistic bits [8, 23, 24, 25]. Prior work has reported sampling throughput gains of up to six orders of magnitude relative to CPU Gibbs sampling and about 5 fold to 18 fold relative to GPU and TPU implementations, together with projected end‐to‐end speedups of 10^2^‐fold to 10^4^‐fold in sampling‐dominated workloads [21]. Device‐level realizations further strengthen this advantage. Compact probabilistic circuits have reported random‐bit generation near 10 MHz with energy near 1 fJ per bit, and spintronic implementations based on magnetic tunnel junctions provide intrinsic stochasticity and nonlinearity with nanosecond switching dynamics and very low operating energy [8, 23, 24, 25].

Taken together, these results identify pIMs as a compelling hardware platform for PQC. For Falcon‐class post‐quantum accelerators, pIMs address the main cost of signing at the levels of power, latency, hardware footprint and entropy efficiency.

## 4 Sampling Quality for Edge Generative Model

Another modern paradigm that can benefit substantially from an efficient, compact and high‐quality Gaussian sampler are edge generative models. Generative models aim to learn a probability distribution over data [5, 6]. Given observations drawn from an unknown distribution, a generative model approximates this distribution with a parametric model such that new samples can be generated that are statistically consistent with the training set. In most modern pipelines, sampling begins from a simple reference distribution, typically Gaussian, and the model progressively transforms this randomness into a structured data instance. Randomness defines the reference measure from which structure emerges and controls both diversity and variability of the generated outputs [45, 46, 47]. To produce semantically targeted samples, generation is conditioned on external signals, such as text descriptions, semantic maps, or latent codes, which constrain the synthesis and steer the output toward desired semantic attributes.

Diffusion models have emerged as a leading framework for controlled generative synthesis because they combine high output quality with stable training and reliable inference [6, 12]. They generate data by progressively transforming random noise into structured content through a learned denoising trajectory. As shown in Figure 4a, sampling begins from an initial latent variable ***z***_0_ drawn from a standard Gaussian distribution, $N\left(0,1\right)$. In semantic communication, generation is conditioned on the received prompt, which is encoded into a latent representation and then used to guide the denoising process through a diffusion transformer. At each iteration, a noise scheduler controls the refinement process by injecting Gaussian noise and applying the learned denoiser, gradually reducing stochasticity while preserving semantic content. The final refined latent is then decoded into an image or video. Because Gaussian noise enters the inference loop at every step, diffusion performance depends directly on the statistical quality of the sampler. The required properties are well defined and include zero mean, correct variance, accurate tail statistics, and sufficient sampling density. Deviations from these conditions can distort the denoising trajectory, reduce output diversity, and introduce visible artifacts [12, 45, 46, 47].

**FIGURE 4:**
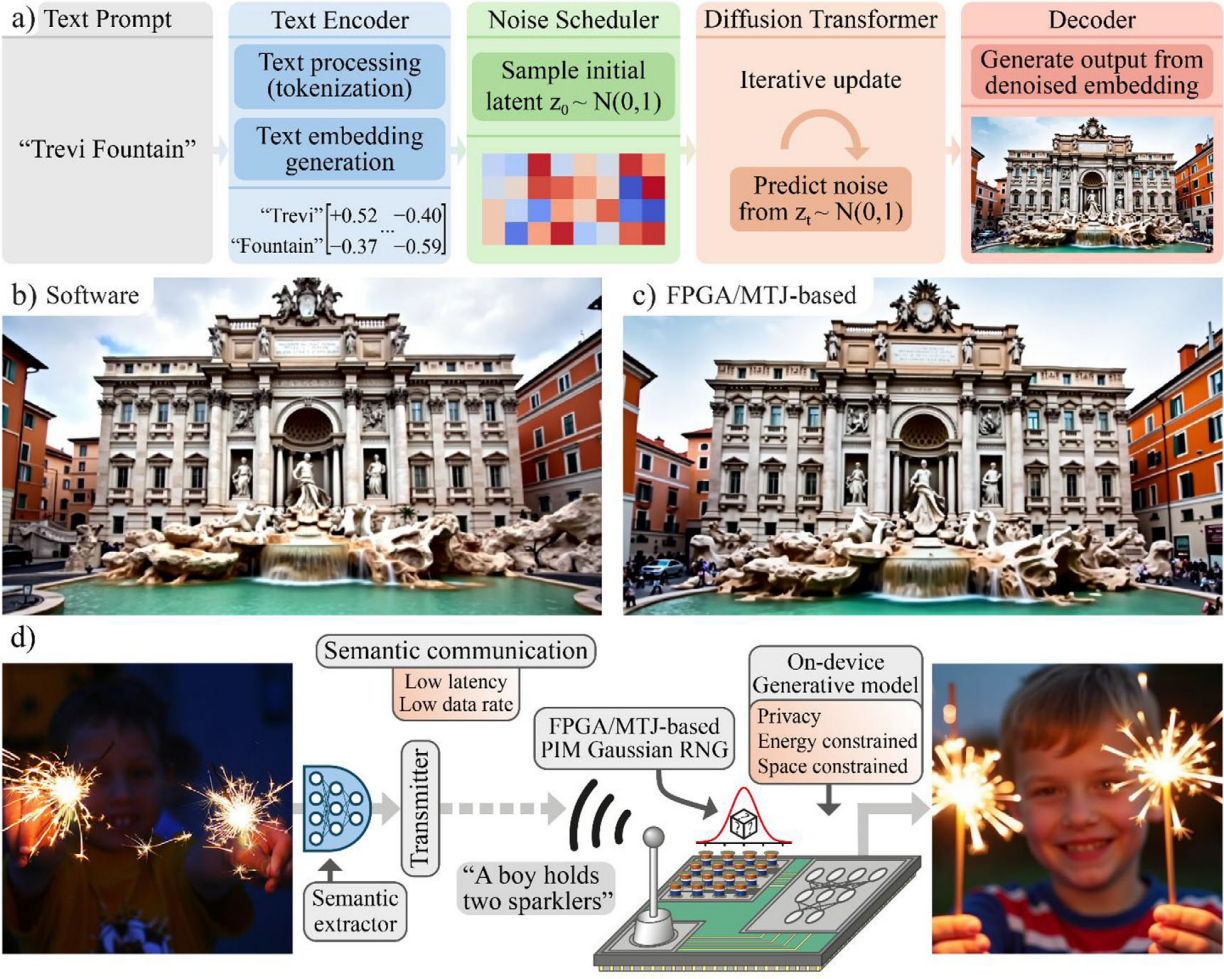
Diffusion model implementation and noise injection points. (a) Schematic of a diffusion model for image and video generation, emphasizing the stages where the Gaussian random matrix is injected by the noise scheduler during iterative denoising and refinement. (b,c) Representative images generated for the text prompt “Trevi Fountain” using (b) a conventional software implementation and (c) an MTJ based implementation, illustrating comparable perceptual quality under identical prompting conditions. (d) Schematic of the semantic communication workflow. A semantic extractor at the transmitter encodes an input image into a compact prompt for low latency, low data rate transmission; an on device generative model at the receiver reconstructs or infers the content under stringent constraints on energy, memory, silicon area, latency, and privacy. Example image and derived prompt are from the Flickr dataset.

To evaluate how Gaussian sampling quality translates into generative performance, we used Open‐Sora [48] to produce the results in Figure 4b,c. Open‐Sora is an open‐source diffusion framework for large scale image and video generation with a modular pipeline comprising text encoding, noise scheduling, diffusion transformers, and decoding, which enables controlled replacement of the noise source without altering the remaining inference path [48]. In our implementation, we modified the noise scheduler to support multiple Gaussian sampling approaches while keeping the same diffusion architecture, scheduler parameters, and model weights. Figure 4b shows an image generated for the text prompt “Trevi Fountain” using the standard PyTorch Gaussian sampler, whereas Figure 4c replaces this noise source with Gaussian samples obtained experimentally from an FPGA/MTJ‐based probabilistic Ising machine. The FPGA/MTJ‐based implementation uses experimentally measured device statistics. Under the same conditions, the FPGA‐only implementation produced no observable difference relative to the software baseline. All other elements of the diffusion pipeline, including model weights, scheduler parameters, and conditioning, are held constant, so that differences can be attributed solely to the noise generator. It can be noted a comparable perceptual quality of the two outputs that as discussed below mirror in different metrics evaluation.

High quality generation at the edge is a key enabler of semantic communication, where transmission is optimized for task relevant meaning rather than raw data [1, 2, 3, 4, 5, 6]. The transmitter compresses high dimensional inputs into compact semantic representations, and the receiver reconstructs the signal or performs inference directly [4, 6]. This reduces data rate and latency while preserving downstream task performance. The advantage is especially relevant in edge systems, where privacy, energy, memory and real time constraints limit conventional transmission and reconstruction, as illustrated schematically in Figure 4d [2, 3, 4, 6]. The main trade‐off is that computational complexity shifts to the receiver, which must regenerate high dimensional content from compact prompts using generative models. Further details on semantic communication are provided in Note S2. Communication efficiency therefore improves, while overall performance becomes increasingly dependent on the quality of the semantic representation and on the efficiency of the receiver hardware.

To assess generation quality in a state‐of‐the‐art diffusion pipeline, we performed a controlled comparison between decoding driven by the standard PyTorch Gaussian sampler and decoding driven by the proposed FPGA/MTJ‐based pIM sampler. We used Open‐Sora [48] with identical model weights, diffusion architecture, scheduler settings, and prompts, changing only the source of Gaussian noise. Each image generation required 147 456 random values. We evaluated two prompting regimes. For the fixed prompt “Trevi Fountain”, we generated 99 images per sampler using the *N = 3, M = 0* pIM configuration and compared them with publicly available reference photographs. For the Flickr evaluation, we randomly selected approximately 500 prompts and generated 10 images per prompt using the software sampler and three pIM configurations. The *N = 3, M = 0* configuration provided the baseline, *N = 4, M = 0* extended the maximum absolute value from 3.5 to 7.5, and *N = 3, M = 0* increased the density of representable values.

Table 2 reports complementary measures of image quality and semantic consistency. Entropy measures global information content, CPBD quantifies perceptual blur, and BLIINDS‐II, BRISQUE, and NIQE assess naturalness and distortion from image statistics [49, 50, 51, 52, 53, 54, 55, 56, 57]. FID measures distribution‐level agreement with reference data, LPIPS measures perceptual similarity, and CLIP distance provides a proxy for text–image alignment. Further details are provided in Note S3. CLIP distance is omitted for the “Trevi Fountain” evaluation because the short prompt admits many visually valid interpretations and therefore provides limited specificity for assessing prompt compliance.

**TABLE 2:** Image‐quality and semantic metrics for Gaussian samplers. Panels (a) and (b) report the Trevi Fountain evaluation using reference images from public datasets. The software baseline uses the standard PyTorch Gaussian sampler, and the pIM result uses N = 3, M = 0. Panels (c) and (d) report the controlled evaluation of approximately 500 Flickr prompts using the software sampler and pIM configurations N = 3, M = 0, N = 4, M = 0, and N = 3, M = 4. Panel a reports entropy, CPBD, BLIINDS‐II, BRISQUE, NIQE, and FID for the Trevi Fountain evaluation; panel b reports LPIPS. Panel c reports the same six image‐quality metrics for the Flickr evaluation; panel d reports LPIPS and CLIP distance. Upward arrows identify metrics for which larger values indicate better performance, and downward arrows identify metrics for which smaller values indicate better performance. Values are means ± standard deviations across generated samples. Bold entries mark the best value within each metric.

a) Image quality							b) Semantic	
	Entropy ↑	CPBD ↑	BLIINDS‐II ↓	BRIQUE ↓	NIQE ↓	FID ↓	LPIPS ↓	
Reference	7.62 ± 0.18	0.71 ± 0.09	10.3 ± 9.5	20.9 ± 8.9	2.42 ± 0.69	−	−	
Software	7.85 ± 0.07	0.29 ± 0.06	44.1 ± 4.5	43.2 ± 5.9	4.40 ± 0.57	0.32 ± 0.02	0.57 ± 0.05	
pIM N=3,M=0	7.84 ± 0.07	0.28 ± 0.06	45.7 ± 5.5	43.8 ± 5.4	4.42 ± 0.56	0.33 ± 0.01	0.56 ± 0.05	

c) Image quality							d) Semantic	
	Entropy ↑	CPBD ↑	BLIINDS‐II ↓	BRIQUE ↓	NIQE ↓	FID ↓	LPIPS ↓	CLIP ↓
Software	7.51 ± 0.33	0.53 ± 0.16	30.4 ± 9.1	32.5 ± 9.6	3.28 ± 0.54	0.19 ± 0.01	0.60 ± 0.03	0.32 ± 0.03
pIM N=3,M=0	7.57 ± 0.43	0.49 ± 0.17	36.3 ± 7.9	36.3 ± 8.4	3.50 ± 0.59	0.19 ± 0.01	0.60 ± 0.04	0.31 ± 0.03
pIM N=4,M=0	7.57 ± 0.42	0.49 ± 0.17	36.3 ± 7.8	36.4 ± 8.3	3.50 ± 0.59	0.15 ± 0.01	0.71 ± 0.07	0.29 ± 0.04
pIM N=3,M=4	7.50 ± 0.33	0.54 ± 0.15	29.9 ± 9.9	32.2 ± 10.0	3.30 ± 0.61	0.16 ± 0.01	0.70 ± 0.07	0.29 ± 0.04

For the fixed prompt, the *N* = 3, *M* = 0 pIM sampler produced metric values within the observed variability of the software baseline. In the Flickr evaluation, extending the tails with *N* = 4, *M* = 0 produced metric‐dependent changes, including lower FID and CLIP distance and higher LPIPS. Increasing the sampling density with *N* = 3, *M* = 4 brought the no‐reference image‐quality metrics and FID closer to the software results, while LPIPS and CLIP distance showed mixed responses. These results show that the pIM sampler supports diffusion‐based generation across different *N*, *M* configurations and that sampling density has a clearer influence on the reported image‐quality metrics than the extension of rare tail values. The dependence on tail range and value density arises from the discretized Gaussian distribution and therefore applies independently of the hardware or software mechanism used to generate the samples.

## 5 Discussion and Conclusions

This work establishes pIMs as a hardware‐native route to Gaussian sampling for post‐quantum cryptography and edge generative inference. State‐of‐art samplers implement discrete Gaussian generation through arithmetic transformations, pseudorandom‐number generation and rejection sampling within fully digital logic [9, 10, 58, 59, 60]. By contrast, the present approach realizes Gaussian sampling directly as a probabilistic hardware primitive. This shift is the central novelty of the study. It replaces a digitally synthesized stochastic routine with a physically grounded sampling mechanism whose variance, discretization and tail coverage can be tuned explicitly through the underlying Ising construction. At the hardware level, the sampling pipeline reduces to multiply and accumulate, nonlinear activation and probabilistic update, which separates the core operations cleanly and enables independent optimization of each block without altering the target statistics.

Relative to state‐of‐the‐art Falcon hardware [9, 10], this reformulation yields a clear reduction in sampler cost. Falcon serves as a representative sampling‐intensive post‐quantum application that demonstrates the applicability of the proposed uniform‐to‐discrete‐Gaussian primitive. The comparison is limited to the sampling stage and does not represent a complete signature‐generation accelerator. In a deployed signer, the sampler is agnostic to the certified uniform random source. At matched sampling precision, the pIM implementation reduces dynamic power by about 4 fold relative to the reported Falcon Gaussian core and by about 8 fold relative to the complete Falcon sampler, while projected ASIC scaling preserves about one order of magnitude advantage under equivalent technology assumptions. The hardware footprint follows the same trend. Relative to the Falcon Gaussian core, the proposed sampler reduces logic usage by about 4.4 fold, and relative to the full sampler the reduction reaches about 8.7 fold, while eliminating DSP consumption entirely. The latency advantage is also clear from an algorithm point of view, since it significantly reduces the required number of random values generated used in state‐of‐art rejection‐based discrete Gaussian routine. By generating natively relevant parameters with a Gaussian distribution frequency, it represents, at the algorithmic level, an latency advantage of about 2 fold for the sampling stage. At the architectural level, sparse pIMs support massively parallel stochastic updates, with reported throughput gains of up to six orders of magnitude relative to CPU Gibbs sampling and about 5 fold to 18 fold relative to GPU and TPU implementations, together with projected end‐to‐end speedups of 100 fold to 10 000 fold in sampling‐dominated workloads [8, 43, 44].

The proposed framework exploits spintronic components for improved performance over prior random‐number delivery schemes. Prior CMOS and FPGA true random‐number generators based on jitter or metastability achieve useful rates, but their energy and area remain significant at the system level [38, 39, 40]. In the present framework, STT‐MTJs serves directly as the entropy‐bearing physical element of the sampler. This reduces the energy of the stochastic core by about two orders of magnitude relative to the corresponding digital block and removes the need to realize randomness and nonlinearity as separate overheads. Prior MTJ work has already shown compact true‐random‐bit generation with NIST‐validated output and projected sub‐picojoule‐per‐bit operation [43, 44]. In this framework, the main system‐level cost is shifted primarily to converters and peripheral interfacing, which makes the next engineering target clear. The main path forward is tighter mixed‐signal integration rather than further optimization of the stochastic core itself.

 In diffusion‐based decoding, the measured samplers reproduce the target Gaussian statistics accurately in the high‐probability region. The residual tail deviations observed in the MTJ case do not produce measurable degradation in image quality or semantic consistency. This point is important for edge semantic communication, where diffusion decoding must reconstruct high‐dimensional perceptual content from compact semantic messages under strict limits on energy, area, and memory traffic. These results are particularly significant for edge semantic communication, where receiver side diffusion decoding must reconstruct high dimensional perceptual content from compact semantic messages under severe limits on energy, area and memory traffic. In this regime, Gaussian sampling is a design constraint. A recent work further indicates that probabilistic generative hardware can reduce energy per generated sample by up to 10^4^ times at GPU comparable performance on binarized Fashion‐MNIST, and lower total energy by about 10 times on CIFAR‐10 through a hybrid architecture that uses a deterministic network that is itself about 10 times smaller [25]. By replacing arithmetic based pseudorandom generation with physically grounded stochastic sampling, the proposed hardware reduces the cost of a central operation in diffusion decoding while maintaining inference quality.

The broader implication is that Gaussian sampling can be treated as an efficient hardware primitive rather than as an expensive auxiliary function. This matters directly for edge generative AI, semantic communication, and cryptographic accelerators, where repeated Gaussian sampling strongly affects both area and energy budgets. By combining competitive footprint, preserved generative fidelity, and large reductions in sampling energy, this work provides a practical route toward compact probabilistic hardware for edge communication paradigms. More generally, it supports a shift in hardware design from deterministic approximation of stochastic processes to direct physical implementation of probabilistic computation.

## 6 Experimental Section/Methods

### 6.1 FPGA Implementation

The FPGA architecture consists of a uniform random number generator (Xoshiro128++), a hyperbolic tangent unit, and two pipelined multi‐operand MAC units [61], where one is dedicated to the local field computation required for the p‐bit updates, and one is used to generate Gaussian random numbers from the p‐bit configuration. The design follows a pipeline‐driven dataflow architecture, explicitly conceived to sustain high throughput, enables partial temporal overlap between local field accumulation and stochastic p‐bit updates, while respecting the inherently sequential update semantics of pIMs. The coupling matrix 𝐽 is stored in FPGA on‐board Block RAM using a memory organization aligned with the pipeline structure, allowing multiple coefficients to be accessed in parallel and injected at different pipeline stages without additional reordering logic. The architecture was synthesized on an AMD ZCU106 board and interfaces with the processing system through an AXI interface for the acquisition of the generated random numbers. The FPGA Register Transfer Language (RTL) is implemented in SystemVerilog and synthesized using Vivado 2023.1, operating at a system clock frequency of 200 MHz. For details about the implementation see the Note S4.

### 6.2 FPGA/MTJ Implementation

The MTJ board consists of 16‐channel digital‐to‐analog converters (DACs, model LTC2688) to drive each MTJ and 16‐channel analog‐to‐digital converters (ADCs, model MAX11131) to sample the output of each MTJ. Both DAC and ADC modules interface with the FPGA (AMD Virtex UltraScale+ VCU118 Evaluation Kit) via serial peripheral interface (SPI) through FMCP‐IPEX connectors. For generating Gaussian distributions using Markov chain Monte Carlo (MCMC)‐based sampling, the p‐bits in each Ising‐based GRNG configuration are updated sequentially. The sequential update ensures each MTJ operates as an independent GRNG. Specifically, during each sampling iteration, the FPGA computes feedback signals for every MTJ cell. The feedback signals are derived from the spin states read by the ADCs and calculated using a MAC operation between the coupling matrix 𝐽 and the spin vectors. The sampled spin vectors are converted to g‐bits, which are then transmitted to a host computer via a user datagram protocol (UDP) ethernet connection. The FPGA RTL is implemented in Verilog and synthesized using Vivado 2023.2. The FPGA operates with a primary system clock of 100 MHz. For further details on the implementation map for the MTJs, see Note S5.

### 6.3 FPGA Resource Utilization and Power Estimation

Resource utilization and power consumption were evaluated using synthesis and implementation reports generated by Xilinx Vivado 2023 for the target FPGA platforms (Xilinx ZCU106 and Virtex UltraScale+ VCU118).

Resource metrics, including CLBs, BRAM, and DSP slices, were extracted from post‐implementation reports to account for placement and routing effects.

Estimation of power consumption was performed using the Vivado power analysis tool, which provides analytical estimates based on the implemented netlist and assumed switching activity. In this work, no application‐specific activity files (SAIF or VCD) were used, and default switching assumptions were adopted. Consequently, the reported power values represent a conservative estimate, corresponding to a near worst‐case operating scenario, and can be interpreted as an upper bound of the actual power consumption.

The above methodology was applied to evaluate the resource utilization and power consumption of the Z‐sampler block of the Falcon architecture on an AMD Xilinx ZCU106 FPGA platform. The design was synthesized out‐of‐context to isolate the contribution of the sampler module and obtain technology‐mapped estimates independent of system‐level integration. In this configuration, the full Z‐sampler implementation occupies 14404 CLBs, 74 DSPs, and no BRAM tiles, with an estimated dynamic power consumption of 641 mW.

A direct comparison with the proposed Gaussian random number generators would not be fully fair, as the Z‐sampler includes additional components beyond the core sampling logic. In particular, the Falcon architecture requires double‐precision floating‐point inputs, leading to the inclusion of floating‐to‐fixed and fixed‐to‐floating conversion units, and employs a ChaCha‐based PRNG to meet cryptographic requirements, which is significantly more complex than generators such as xoshiro that we employed in the FPGA‐based implementation [62]. To enable a more meaningful comparison, the Gaussian sampling core was further isolated by excluding the cryptographic PRNG and data conversion blocks. Under this configuration, the Gaussian sampling module alone requires 6947 CLBs, 67 DSPs, and no BRAMs, with an estimated dynamic power consumption of 316 mW.

The small differences in the resources utilization with respect to results reported in ref. [9] can be attributed to the selection of a different board (ZCU106 instead of ZCU 104). The same methodology was consistently applied to both the Falcon‐based sampler and the proposed Gaussian random number generators, which were synthesized and evaluated in isolation. This isolated configuration constitutes the functionally matched baseline used throughout for like‐for‐like comparison, since it performs the same task as the proposed sampler, mapping uniform random numbers into discrete Gaussian samples, without the cryptographic PRNG and format‐conversion overhead present in the complete Z‐sampler. This ensures a fair comparison of relative efficiency and enables the assessment of power consumption independently of application‐specific workloads, under a conservative and near worst‐case operating scenario.

## Author Contributions

**Davi Rodrigues**: conceptualization, investigation, methodology, validation, supervision, Writing – review and editing, Writing – original draft, software, visualization, data curation, formal analysis. **Andrea Grimaldi**: investigation, methodology, validation, writing – review and editing, visualization, software. **Deborah Volpe**: investigation, methodology, validation, writing – review and editing, visualization, software. **Youwei Bao**: investigation, validation, methodology, visualization, writing – review and editing, software. **Shuhan Yang**: writing – review and editing, investigation, validation, methodology, visualization, software. **Eleonora Grassucci**: investigation, writing – review and editing, validation, methodology, software, data curation. **Giulio Siracusano**: investigation, validation, methodology, software, data curation. **Massimo Chiappini**: funding acquisition, writing – review and editing, supervision, project administration. **Mario Carpentieri**: project administration, funding acquisition, supervision, writing – review and editing, resources. **Hyunsoo Yang**: writing – review and editing, funding acquisition, project administration, supervision, resources. **Giovanni Finocchio**: writing – review and editing, conceptualization, funding acquisition, project administration, supervision, resources, formal analysis.

## Funding

This work was supported by Project PRIN 2020LWPKH7 “The Italian factory of micromagnetic modeling and spintronics” (IT‐SPIN). D.V. acknowledge funding from the project PON Capitale Umano (CIR_00030) funded by the Italian Ministry of Universities and Research (MUR), and D.D. n. 47 del 20 febbraio 2025 – “Assunzione di ricercatori internazionali post‐dottorato”, CUP: D53C25000630007 (D.V.). H.Y. is supported by the Ministry of Education, Singapore, under Tier 1 (A‐8004125‐00‐00, A‐8004460‐00‐00) and National Research Foundation Singapore (NRF‐T‐CRP‐2025‐0001, NRF‐CRP32‐2024‐0003). DR, AG, DV. MC, and GF are with Petaspin team and thank the support of the PETASPIN association (www.petaspin.com).

## Conflicts of Interest

The authors declare no conflicts of interest.

## Supporting information




**Supporting File**: advs78214‐sup‐0001‐SuppMat.docx.

## Data Availability

All data supporting the findings of this study will be made available from the corresponding author upon reasonable request.
