# Supplementary material for: Hardware‐Native Gaussian Sampling With Probabilistic Ising Machines for Modern Communication Paradigms
Source: Adv Sci (Weinh). 2026 Oct 11:e78214. Online ahead of print. doi: 10.1002/advs.78214 (PMC13655251; doi:10.1002/advs.78214)
Supplement: Supplementary file 1 [file ADVS-9999-e78214-s001.docx]

Supporting Information

Supplementary Notes - Hardware-native Gaussian sampling with probabilistic Ising machines for Modern Communication Paradigms

Davi Rodrigues^1,*^, Andrea Grimaldi^1^, Deborah Volpe^2^, Youwei Bao^3^, Shuhan Yang^3^, Eleonora Grassucci^4^, Giulio Siracusano^5^, Massimo Chiappini,^2^ Mario Carpentieri^1^, Hyunsoo Yang^3,*^, and Giovanni Finocchio^5,*^

^1^Department of Electrical and Information Engineering, Politecnico di Bari, 70126 Bari, Italy

^2^Istituto Nazionale di Geofisica e Vulcanologia, 00143 Rome, Italy

^3^Department of Electrical and Computer Engineering, National University of Singapore, Singapore 117583, Singapore

^4^Department of Information Engineering, Electronics, and Telecommunications, Sapienza University of Rome, 00184, Rome, Italy

^5^Department of Mathematical and Computer Sciences, Physical Sciences and Earth Sciences, University of Messina, 98166, Messina, Italy

*corresponding authors: [davi.rodrigues@poliba.it](mailto:davi.rodrigues@poliba.it), [eleyang@nus.edu.sg](mailto:eleyang@nus.edu.sg), gfinocchio@unime.it

**NOTE 1 – STATISTICAL VALIDATION OF THE GAUSSIAN SAMPLING**

We evaluated the statistical quality of Gaussian samples generated by the software, digital, and experimental Ising implementations using three complementary metrics and three update protocols. The first protocol applies one standard Ising update before recording each sample. The second applies three consecutive updates, with the first performed using 0.1 times the effective field. This reduced field corresponds to a higher effective temperature, which promotes state mixing and reduces temporal correlations between successive samples. The third protocol introduces an additional sign bit beyond the *N+M* bits used to encode the magnitude. This bit is uncoupled from the Ising network and is independently sampled with equal probabilities of 0 and 1. It therefore assigns the sign of each sampled value without introducing temporal correlations. For simplicity, this protocol was evaluated only in the software implementation. Its operation depends solely on an independent unbiased bit and can therefore be applied equivalently to the digital and experimental implementations.

Here, the Kullback–Leibler divergence quantifies agreement between the sampled probability mass function and the target discretized Gaussian distribution. The autocorrelation function measures temporal dependence between successive samples and its effect on the effective sample size. Quantile–quantile plots assess agreement across the distribution, including its tails. Together, these analyses separate deviations in the stationary distribution from temporal correlations introduced by the update dynamics.

*Convergence to the target distribution*

Figure S.1 reports the Kullback-Leibler divergence as a function of sample count for four encodings. The three implementations approach a configuration-dependent divergence floor. This residual value arises from representing a continuous Gaussian distribution on a finite discrete support. For N = 3, increasing M from 0 to 2 increases the sampling density and lowers the asymptotic divergence from approximately 10^-3^ to approximately 10^-4^. The software and digital implementations reach comparable floors, while the experimental curves remain modestly higher for the N = 3 configurations over the evaluated sample range. These differences quantify implementation-specific sampling error and do not indicate a change in the encoded support.

The N = 4, M = 0 configuration shows a distinct convergence transient when each spin is updated once per sweep. The solid curves remain at high divergence for substantially more samples and exhibit pronounced fluctuations before approaching the discretization floor. Updating each spin three times per sweep removes this prolonged transient in all three implementations. In the software implementation, supplementing a single spin update with an uncorrelated spin value produces a similar improvement. The dependence on update protocol indicates that the slow convergence originates from Markov-chain mixing for this encoding.

**Figure S.1. Convergence of the sampled distributions.** Kullback-Leibler divergence between the sampled probability mass function and the target discretized Gaussian distribution as a function of sample count for (a) N = 3, M = 0, (b) N = 3, M = 1, (c) N = 3, M = 2, and (d) N = 4, M = 0. Blue, green, and orange curves correspond to the software, digital, and experimental implementations, respectively. Solid curves show one update per spin per sweep, and dashed curves show three updates per spin per sweep. The blue dash-dotted curve shows the software implementation with one update per spin supplemented by an uncorrelated spin value. Both axes in panel d and the divergence and sample-count axes in panels a-c use logarithmic scales. Increasing M lowers the discretization-limited divergence floor. For N = 4, M = 0, the single-update protocol converges slowly, whereas the additional-update and decorrelated-spin protocols approach the floor at lower sample counts.

*Temporal correlations and mixing*

The autocorrelation analysis in Figure S.4 identifies the origin of the delayed convergence. For N = 3, M = 0, 1, and 2, one update per spin produces positive short-range correlation that decays progressively with lag and approaches zero within the displayed 50-lag interval. Three updates per spin reduce the correlation to near zero after the first lag. The uncorrelated-spin protocol produces the same qualitative behavior in software.

For N = 4, M = 0, the one-update curves remain close to 0.7 throughout the full lag window. This persistent correlation explains why additional samples provide limited new information and why the corresponding Kullback-Leibler divergence converges slowly. Three updates per spin cause the correlation to decay to approximately zero within about ten lags. Adding an uncorrelated spin in the software implementation suppresses the correlation within the first lag. The agreement among the software, digital, and experimental trends links the observed behavior to the encoded sampling dynamics.

*Quantile agreement*

Figure S.2 compares empirical and theoretical quantiles for the four encodings. The staircase structure reflects the finite set of values available in each discretized representation. Increasing M increases the number of representable levels, yielding a denser quantile relation for N = 3, M = 1 and N = 3, M = 2. Across the central region, the curves from the software, digital, and experimental implementations largely overlap and follow the expected monotonic quantile relation. Small separations occur mainly near the distribution endpoints, where observations are less frequent and quantile estimates are more sensitive to finite sampling.

The update protocols produce closely aligned quantile curves once sufficient samples are collected. This observation is consistent with the Kullback-Leibler analysis, which shows convergence toward the same discretization-limited distribution. The principal effect of the update protocol is therefore the rate at which effectively independent samples are obtained, particularly for N = 4, M = 0. The combined results show that sampling fidelity is controlled by both the resolution of the encoded distribution and the mixing efficiency of the spin-update schedule.

**Figure S.2. Agreement between empirical and theoretical quantiles. Q**uantile-quantile plots for (a) N = 3, M = 0, (b) N = 3, M = 1, (c) N = 3, M = 2, and (d) N = 4, M = 0. The horizontal axis gives the theoretical quantiles of the target discretized Gaussian distribution, and the vertical axis gives the corresponding sample quantiles. Colors and line styles follow Fig. S.2. The staircase profiles arise from the discrete output levels. Increasing M increases the level density, as seen by the progressively finer steps from panels (a) to (c). The close overlap among implementations and update protocols indicates consistent quantile coverage, with the largest visible differences confined to sparsely sampled endpoint values.

**NOTE 2 - SEMANTIC COMMUNICATION**

Semantic communication revisits Shannon’s semantic and effectiveness levels by optimizing wireless transmission for meaning preservation and task success, rather than for exact symbol recovery. [1,4,6] In this paradigm, the transmitter extracts task relevant semantics from high dimensional data, for example as latent variables or a textual or structured prompt, and transmits only this compact representation. The receiver then reconstructs an interpretable signal or directly performs inference, so that moderate symbol level distortions are acceptable provided semantic fidelity and downstream performance are preserved. A representative implementation is shown in Fig. S.3. The transmitter applies a semantic extractor to compress an input image into a short prompt for low latency, low data rate transmission, while the receiver employs an on device generative model to reconstruct or infer content under tight constraints on energy, memory footprint, silicon area, privacy, and real time execution. Prompt length provides a direct rate fidelity control knob. Starting from the original image in Fig. S.1(a), we generated three prompts of increasing length using a commercial semantic extractor and provided them to Open-Sora, an open-source diffusion model implemented with the architecture proposed in this manuscript in which noise is generated by pIMs. The resulting reconstructions, shown in Fig. S1(b–d), become progressively more detailed and semantically specific as prompt length increases, highlighting the underlying rate–fidelity trade-off. We considered the following prompts: (b) “A boy holds two sparklers.” (c) “A child in a dim blue room holds two lit sparklers toward the camera, creating bright golden sparks and light trails, wearing a yellow T shirt.” (d) “Photograph in low light with a strong blue cast in the background, suggesting dusk or an indoor setting. A Caucasian child is centered and slightly out of focus behind the foreground lights, smiling broadly and showing the front teeth while extending both arms toward the camera. The child holds a lit sparkler in each hand, with the sparklers closer to the lens than the face. The sparklers are in sharper focus and dominate the foreground, producing intense white gold cores with many orange yellow sparks radiating outward, creating short streaks and curved light trails across the frame. The left sparkler appears near the upper left and the right sparkler near the upper right, giving a balanced composition. The child wears a yellow T shirt with a large chest graphic showing a soccer ball, with the words BIO VIST written across it in yellow lettering. The overall scene has high contrast between the bright sparklers and the dark background, shallow depth of field, and bokeh like spark highlights, conveying a joyful celebratory moment.” These prompts were generated using commercially available semantic extractors. Shorter prompts reduce latency and energy consumption but leave fine visual details underspecified, whereas longer prompts improve reconstruction fidelity at higher communication cost. This workflow shifts system complexity from channel coding to semantic representation and generative reconstruction, thereby tying communication performance to the statistical structure and inductive biases of the underlying models, while simultaneously relocating the dominant burden to the receiver and increasing the demand for energy and area efficient hardware capable of regenerating high dimensional data from compact semantic messages.

**Figure S3. Data transmission within semantic communication.** (a) Original image. (b-d) Generated images obtained with increasing prompt length, illustrating the communication and fidelity trade off: shorter prompts reduce transmitted information but may omit fine grained attributes, whereas longer prompts improve semantic specificity at higher communication cost.

**NOTE 3 - METRICS FOR IMAGE QUALITY ANALYSIS**

The metrics adopted in this work can be divided into two groups. No-reference (NR) indices assess each generated image in isolation, without access to a pristine original, while reference-based metrics quantify how closely the generated outputs match a reference image set in terms of distributional statistics or perceptual similarity.

**No-reference metrics**

*Entropy*

Shannon entropy  [59] captures the average information content of an image by quantifying the uncertainty in its pixel-intensity distribution. For a colour image, the entropy is computed on its luminance representation. Given a normalised intensity histogram *p*(*i*), the entropy is:

$H=-\sum_{i=0}^{L-1} p\left(i\right)\cdot\log_{2}p\left(i\right)$ (S.1)

where the sum runs over all intensity levels. Images with richer tonal variation yield higher entropy, whereas flat or narrow-histogram images produce lower values.

*Cumulative Probability of Blur Detection (CPBD)*

CPBD  [55] quantifies perceived image sharpness by modelling how the human visual system detects blur at individual edges. At each detected edge *i*, the probability of perceiving blur is *P*_BLUR_(*i*):

$P_{BLUR}\left(i\right)=1-e^{\left(-\left|w_{i}/w_{JNB\left(C_{i}\right)}\right|^{\beta}\right)}$ (S.2)

where *w*ᵢ is the measured edge width, *C*ᵢ is the local contrast, *w*_JNB(Cᵢ)_ is the just-noticeable-blur width for that contrast level — a threshold derived from psychophysical experiments — and *β* is a shape parameter governing the detection curve. The overall CPBD score corresponds to the fraction of edges in the image for which *P*_BLUR_ remains below a perceptibility threshold; higher scores therefore indicate sharper images.

*BLind Image Integrity Notator using DCT Statistics (BLIINDS-II)*

BLIINDS-II  [54] builds a natural scene statistics (NSS) model in the discrete cosine transform (DCT) domain. Local image patches are transformed into DCT coefficients, which are then fitted to a generalised Gaussian distribution (GGD) characterised by a shape parameter α and a variance σ². These fitted parameters, extracted at multiple spatial scales, serve as quality-aware features. A Bayesian inference framework maps the feature vector to a single quality score, with lower values indicating better perceptual quality.

*Blind/Referenceless Image Spatial QUality Evaluator (BRISQUE)*

Rather than transforming images into a frequency domain, BRISQUE  [52] works directly on pixel intensities through mean-subtracted contrast-normalised (MSCN) coefficients. At each pixel (*i*, *j*), these are defined as:

$\hat{I}\left(i,j\right)=\left[I\left(i,j\right)-\mu\left(i,j\right)\right]/\left[\sigma\left(i,j\right)+C\right]$ (S.3)

where *I*(*i*, *j*) is the pixel intensity, *μ*(*i*, *j*) and *σ*(*i*, *j*) are the local mean and standard deviation obtained via a Gaussian weighting window, and *C* is a small constant that prevents division by zero. This normalisation removes local luminance and contrast, exposing the underlying statistical structure of the image. In undistorted natural images, MSCN coefficients closely follow a Gaussian distribution; distortions shift this distribution in characteristic ways. The MSCN histogram is fitted to a GGD, and pairwise products of neighbouring MSCN coefficients along four orientations (horizontal, vertical, and both diagonals) are fitted to an asymmetric GGD (AGGD), yielding four parameters per orientation: shape, distribution mean, and separate left and right variance terms. Taken together at two spatial scales, these form a 36-dimensional feature vector that is mapped to a quality score through support vector regression trained on human opinion scores. Lower BRISQUE values correspond to higher perceptual quality.

*Natural Image Quality Evaluator (NIQE)*

NIQE  [53] differs from BRISQUE in a fundamental way: it is entirely opinion-unaware and requires no exposure to distorted images or subjective quality scores during training. It extracts the same MSCN-based spatial features described above from local patches, but instead of learning a regression model, it builds a multivariate Gaussian (MVG) reference model, 𝒩(ν₁, Σ₁), from a corpus of pristine natural images. The quality of a test image is then measured as the distance between its own MVG feature model, (ν₂, Σ₂), and the pristine reference:

$NIQE=\sqrt{\left[\left(v_{1}-v_{2}\right)^{T}\cdot\left(\left(\sum_{1}+\sum_{2}\right)/2\right)^{-1}\cdot\left(v_{1}-v_{2}\right)\right]}$ (S.4)

where ν₁, Σ₁ and ν₂, Σ₂ are the mean vectors and covariance matrices of the pristine and test-image feature distributions, respectively. Lower NIQE scores indicate that the test image is statistically closer to the natural-image reference and therefore of higher perceptual quality.

**Reference-based metrics**

*Fréchet Inception Distance (FID)*

FID  [51] operates at the level of image distributions rather than individual images, making it sensitive to both the quality and the diversity of a generated set. Real and generated images are passed through a pre-trained Inception-v3 network [50], and the 2048-dimensional activations from its penultimate pooling layer are modelled as multivariate Gaussians. The FID is defined as the squared Fréchet (Wasserstein-2) distance between these two Gaussians:

$FID=\left\|\mu_{r}-\mu_{g}\right\|_{2}^{2}+Tr\left(\sum_{r}+\sum_{g}-2\left(\sum_{r}\cdot\sum_{g}\right)^{1/2}\right)$ (S.5)

where (*μ*_r_, Σ_r_) and (*μ*_g_, Σg) are the mean vectors and covariance matrices of the real and generated feature distributions, ‖·‖₂ is the Euclidean norm, *Tr*(·) is the matrix trace, and (Σ_r_ Σ_g_)^1/2^ is the matrix square root of the covariance product. A lower FID indicates closer distributional agreement between generated and reference images.

Learned Perceptual Image Patch Similarity (LPIPS)

LPIPS  [49] measures the perceptual distance between two individual images, *x* and *x*_₀_, by comparing their internal representations within a pre-trained deep network such as AlexNet [60]. Feature activations are extracted at *L* intermediate layers, unit-normalised in the channel dimension, and compared as

(S.6)

where denotes the channel-normalised activation at layer *l* averaged over spatial dimensions, and *w*_l_ is a learned per-channel weight vector calibrated against human perceptual judgements from the Berkeley-Adobe Perceptual Patch Similarity dataset. Lower LPIPS values indicate that two images are perceptually more similar.

**NOTE 4 -FPGA ARCHITECTURE**

Figure S.4(a) shows the proposed pipelined architecture for Gaussian random number generation based on the probabilistic Ising Machine (pIM). The architecture is designed as a pipeline-driven dataflow architecture in which memory organization, arithmetic pipeline structure, and update scheduling are optimized to sustain high throughput while following the sequential semantics of probabilistic Ising dynamics. Instead of adapting the pipeline to the design, the architecture is explicitly conceived to support the pipeline flow, enabling partial temporal overlap between computation stages that would otherwise be strictly serialized by data dependencies.

**Figure S.4. Pipelined Gaussian random number generator for FPGA based on Probabilistic Ising Machines** (a) Hierarchical view of the architecture. b) Operations scheduling of the first p-bits update, which can be fully pipelined due to the absence of data dependencies. c) Operations scheduling of the subsequent p-bit updates, which require sequential execution because of inter-update dependencies.

The overall computation is structured around two main phases that naturally arise in the pIM update rule: Phase I, corresponding to the evaluation of the local field associated with each p-bit, and Phase II, corresponding to the stochastic update of the p-bit state. In a conventional implementation, these update of two p-bits is strictly sequential, as the local field of the a p-bit is evaluate considering the state of all the others, introducing strong data dependencies. On the other hand, the proposed architecture exploits a pipelined formulation that partially overlaps in time the evaluation of a p-bit update with the update of the previous p-bit, significantly increasing effective throughput.

The computation of the local field relies on the accumulation of weighted contributions defined by the coupling matrix $\boldsymbol{J}$, and it is computed as $I_{i}\left( t \right)=\beta\left( \sum_{j} J_{ij}m_{j} \right)$. The $\boldsymbol{J}$coefficients are stored in FPGA on-board Block RAM (BRAM) using a multi-row, paged memory organization to increase the memory bandwidth and to align with the structure of the arithmetic pipeline. Differently from conventional memory layouts, where the $\boldsymbol{J}$ matrix is stored in a row-wise or column-wise ordered fashion, the proposed design adopts a pipeline-aware memory mapping. In this architecture, each BRAM row does not correspond to a single logical row of the $\boldsymbol{J}$ matrix. Instead, it contains exactly the set of coefficients required by the first adder tree at a specific stage of the local field computation.

This organization reflects a key architectural insight: because the multi-operand adder is capable of accepting new operands not only at its input but also at intermediate pipeline stages, the coefficients of a logical row of $\boldsymbol{J}$ are consumed at different times during the pipeline traversal. Consequently, storing the matrix in a strictly ordered format would lead to underutilization of memory bandwidth or require additional buffering and reordering logic. On the other hand, the proposed paging strategy distributes the coefficients of each logical row of $\boldsymbol{J}$ across multiple BRAM rows, arranged according to the exact cycle in which each coefficient must be injected into the adder tree. This time-aligned memory layout enables multiple coefficients to be fetched in parallel and injected directly into the appropriate pipeline stages, avoiding stalls and minimizing control overhead.

The local field accumulation itself is performed by a pipelined multi-operand adder, whose structure follows and improve the approach introduced in Ref.  [61]. Differently from conventional adder trees, which accept operands only at their input stage, this adder is explicitly designed to support in-flight operand insertion at multiple pipeline levels. This capability allows newly updated p-bit contributions to be included into the accumulation process while previous partial sums are still propagating through the pipeline. As a result, the architecture can accommodate data dependencies without reverting to full serialization, maintaining high pipeline occupancy even during dependent update phases.

At the beginning of each generation, all p-bit contributions are known, and the local field evaluation proceeds in a fully pipelined manner, achieving maximum throughput. As soon as a partial sum reaches the final stage of the adder tree, the corresponding local field value becomes available and is immediately processed by the update logic. The hyperbolic tangent activation function is implemented using a carefully optimized combination of linear approximation, LookUp Tables (LUTs), and saturation, striking a balance between numerical accuracy and hardware efficiency. The activation output is then compared with a pseudo-random number generated by a Xoshiro128 [62] architecture, producing the updated p-bit state according to the probabilistic update rule. The subsequent p-bit updates exploit the same pipeline. Newly computed contributions are injected into the adder tree at the appropriate pipeline stage, allowing accumulation and update operations to proceed concurrently.

In addition to the local field computation, the architecture integrates a second pipelined adder tree (Adder tree 2) dedicated to the generation of the effective gaussian random number through the p-bits state. Every three complete p-bit update cycles, this block accumulates a selected subset of coefficients with signs determined by the current p-bit configuration. This accumulation process is fully decoupled from the main update pipeline and can proceed entirely in parallel with p-bit updates.

The operation scheduling of the two adder trees is illustrated in Fig. S.4(b) and Fig. S.4(c) and highlights the adaptability of the proposed pipeline mechanism to different dependency scenarios. During the first complete p-bit update (Fig. S.2(b)), all updates are independent, allowing the pipeline to operate in a fully pipelined mode with no stalls. During subsequent updates (Fig. S.4(c)), data dependencies enforce a sequential update order. In this case, the evaluation of the new local field is delayed by a number of clock cycles equal to the evaluation latency plus one additional cycle to satisfy dependency constraints. Even under these conditions, the pipeline remains highly utilized, and the relative benefit of the proposed approach increases with the number of p-bits involved.

Overall, the architecture proves how careful co-design of memory layout, arithmetic pipeline, and update scheduling can overcome the apparent incompatibility between sequential probabilistic dynamics and high-throughput hardware execution. The design is fully parameterizable, with the number of p-bits $N$, $M$ and the number of pipeline stages exposed as configurable parameters.

**NOTE 5 - SPIN-TRANSFER-TORQUE MAGNETIC TUNNEL JUNCTION (STT-MTJ) BASED PROBABILITIS ISING MACHINE**

*STT-MTJ fabrication and characteristics*

STT-MTJs were fabricated with the following film stack: substrate (Si substrates with a 300 nm thermal oxide layer)/[W (3)/Ru (10)]₂/W (3)/Pt (3)/Co (0.25)/Pt (0.2)/[Co (0.25)/Pt (0.5)]5/Co (0.6)/Ru (0.85)/Co (0.6)/Pt (0.2)/Co (0.3)/Pt (0.2)/Co (0.5)/W (0.3)/CoFeB (0.9)/MgO (0.85)/CoFeB (1.2)/W (0.4)/CoFeB (0.8)/MgO (0.7)/Ta (3)/Ru (7)/Ta (5) (thicknesses in nanometers). The MTJ pillars, with a nominal diameter of approximately 90 nm, were defined by electron-beam lithography.

The fabricated STT-MTJs exhibit an antiparallel state resistance R_AP_ of ~5 kΩ, and a parallel state resistance R_P_ of ~2.5 kΩ, with TMR around 100%. The distribution of the thermal stability factor is shown in Figure S.3(a). The write-voltage pulse amplitudes for the 256 MTJs are within the range of 0.6–1.4 V, as shown in Figure S.3(b). Each write operation uses a pulse width of approximately 4 μs. The MTJ state is read immediately after the write pulse, and no separate read-voltage pulse is applied. A 4 μs pulse is used to reset the MTJ. Therefore, one reset-perturb cycle takes approximately 8 μs, corresponding to a repetition rate of 125 kHz.

*System calibration*

In our system, the mapping from the algorithmic Ising parameters (*J*_ij_, *h*_i_) to the physical bias applied to each MTJ is implemented through the probabilistic-bit (p-bit) representation and the experimentally calibrated switching-probability response of each MTJ cell.

For the *i*-th p-bit *s*_i_, its input is calculated from the Ising parameters and the current states of the other p-bits as (we assume here the inverse temperature term to be 1 for simplicity): *I*_i_ = Σ_j_ *J*_ij_ *s*_j_ + *h*_i_. The probability of obtaining *s*_i_ = +1 follows the standard sigmoid response: P_(si = +1)_ = 1 / [1 + exp(−*I*_i_)].

Therefore, the remaining step is to quantitatively map the algorithmic input *I*_i_ to the physical input-voltage pulse V_in,i_ applied to the corresponding MTJ cell. This mapping is obtained experimentally for each MTJ as follows:

1. Characterization of each MTJ cell

We fix the voltage pulse width and sweep the voltage pulse amplitude (V_in_) over the operating range. At each V_in_ value, 10,000 repeated switching trials are performed for each MTJ cell. The switching probability *p*_P_is obtained from the fraction of trials producing the +1 spin state.

2. Cell-wise calibration

The measured switching-probability curve of each MTJ is fitted using: *p*_P,i_(V_in_) = 1 / {1 + exp[−σ_i_(V_in_ − μ_i_)]}, where σ_i_ represents the slope and μ_i_ represents the voltage offset of the *i*-th MTJ probabilistic switching response. These two experimentally extracted parameters account for device-to-device variations.

3. Mapping from the Ising parameters to the physical voltage

Defining the normalized p-bit input as: *I*_i_ = σ_i_(V_in,i_ − μ_i_), the experimentally measured response is transformed into the standard sigmoid: *p*_P(_*_I_*_i)_ = 1 / [1 + exp(−*I*_i_)]. Consequently, during Ising-machine operation, the physical voltage applied to each MTJ is calculated as: V_in,i_ = *I*_i_ / σ_i_ + μ_i_, or equivalently, V_in,i_ = (Σ_j_ *J*_ij_ s_j_ + *h*_i_) / σ_i_ + μ_i._

The statistic distribution of the extracted σ and μ is shown in Figure S.3(c-d). This cell-wise calibration compensates for device-to-device variations.

*Power consumption evaluation*

The MTJ power consumption was estimated by adding the power consumption in the reset and perturb phase. P = I^2^(R_AP_+R_P_), where I (~50 µA) is the average switching current and R_AP_ (~5k Ohm) and R_P_ (~2.5k Ohm) are the MTJ resistance in antiparallel state and parallel state, respectively. Thus, the reported 0.02 mW represents the estimated dynamic device power associated with one reset-perturbation operation of a single MTJ.

The sensing, biasing, and readout contributions are not included in this MTJ value, as these peripheral-circuit contributions are accounted for separately under the DAC + ADC components in Table 1 of main text.
